# Quantifying agonistic interactions between group-housed animals to derive social hierarchies using computer vision: a case study with commercially group-housed rabbits

**DOI:** 10.1038/s41598-023-41104-6

**Published:** 2023-08-29

**Authors:** Nusret Ipek, Liesbeth G. W. Van Damme, Frank A. M. Tuyttens, Jan Verwaeren

**Affiliations:** 1 Department of Data Analysis and Mathematical Modelling, Ghent University, Coupure Links 653, 9000 Gent, Belgium; 2 Animal Sciences Unit, ILVO, Scheldeweg 68, 9090 Melle, Belgium; 3 Department of Veterinary and Biosciences, Faculty of Veterinary Medicine, Ghent University, Salisburylaan 133, 9820 Merelbeke, Belgium

**Keywords:** Animal behaviour, Computational science

## Abstract

In recent years, computer vision has contributed significantly to the study of farm animal behavior. In complex environments such as commercial farms, however, the automated detection of social behavior and specific interactions between animals can be improved. The present study addresses the automated detection of agonistic interactions between caged animals in a complex environment, relying solely on computer vision. An automated pipeline including group-level temporal action segmentation, object detection, object tracking and rule-based action classification for the detection of agonistic interactions was developed and extensively validated at a level unique in the field. Comparing with observations made by human observers, our pipeline reaches 77% precision and 85% recall using a 5-min tolerance interval for the detection of agonistic interactions. Results obtained using this pipeline allow to construct time-dependent socio-matrices of a group of animals and derive metrics on the dominance hierarchy in a semi-automated manner. Group-housed breeding rabbits (does) with their litters in commercial farms are the main use-case in this work, but the idea is probably also applicable to other social farm animals.

## Introduction

The study of behavior, especially specific social behaviors, has always been laborious and time-consuming. The emergence of new monitoring technologies has not significantly changed this: animal behavior is still studied using human observation and interpretation of social interactions between animals. For group-housed animals living in pens, the limited size of the pens facilitates the observation process but continuous behavior monitoring presents challenges. Dedicated scan sampling or focal sampling designs are regularly used to reduce the workload but these reduce the temporal resolution of the observations and can introduce bias and create sampling variability, potentially impairing the validity of the study^1^. Automated monitoring systems can help to overcome the aforementioned shortcomings, and the use of video recording analyzed by computer vision algorithms has often been suggested as a cost-efficient and non-invasive technology^2^. This technology can be used to study animal behavior at a research scale and can be scaled up to study behavior on farm. Computer vision has a potential advantage of eliminating the subjectivity of a human observer^3^. These benefits have led to the development of a plethora of computer vision models for detecting a variety of behaviors for the most agriculturally relevant species^4–6^. Despite the widespread occurrence of hierarchy formation, few studies have used computer vision to study this process. In the present study the automated detection of (dyadic) relationships between farm animals in complex environments was measured using computer vision, with a focus on the detection of agonistic interactions. We show that modern computer vision allows for automated detection of agonistic interactions (such as fights or chasing events) between animals. To the best of our knowledge, this is the first mention of detection and aggregation of all these interactions over a prolonged period of time with the aim of deciphering the dominance hierarchies of commercially group-housed animals. The proposed approach has been extensively validated with observations made by human observers at several levels. Our approach allows continuous automated monitoring of the dominance structure of a group of animals, offering a way to gain insight into the dynamics of hierarchy formation with unprecedented temporal detail. As a case study, we use commercially group-housed rabbits, but the proposed approach can probably be extended to other species as well.

Computer vision is gaining popularity for livestock monitoring and management. Current applications include early detection of diseases, detection of lameness, estimation of weight and even social behavior monitoring of animals^2,7^. Recent advances in the field of computer vision, particularly the use of deep learning, have boosted the robustness of vision-based monitoring systems in complex environments^8,9^. The vast majority of these applications focus on non-interactive behaviors such as drinking, hanging and rearing^10,11^. Detection of agonistic interactions and the identification of individual animals creates a number of computer vision challenges. First and foremost, all of the recent work on animal behavior detection relies on data-hungry deep neural networks that rely on an enormous amount of training material. The detection of agonistic interactions is but one example of the more general problem of action detection in video. End-to-end trainable models such as channel-separated convolutional networks^12^ represent an elegant solution. However, for training, such models require a large number of manually annotated video clips containing one agonistic interaction each, but such a set of video clips is not publicly available. Given the effort required to collect such data in sufficient quantities, it is unlikely that enough publicly available data will be available soon. However, data collection for more simple, individual behaviors is often easier. As an example, for pigs, distinguishing between four different postures (standing, lying on the stomach, lying on the side, and exploring) can be done quite accurately by solving an image classification problem obtaining 92.45% accuracy^13^. In that case, training material is rather easy to collect, and typically consists of a few hundred hand-labeled frames containing one animal each in a static lying or standing position. Detection of agonistic interactions requires much more data, however^14^. We solved this problem using a hybrid approach. First, we developed a dedicated tracker that tracks the animals during fighting/fleeing events. Second, the dominant and submissive animal (according to that interaction) are distinguished based on the detected movement patterns. The tracking of the individual animals is inherently linked to the detection and identification of the animals over time, adding a temporal dimension to the spatial analysis. Numerous established methodologies are available for multiple object tracking, from Kalman filters^15^ to deep learning-based algorithms^16^. Here we used problem-specific engineered trackers. Based on the limited and fixed number of animals in the cage, the movement patterns of each individual could be extracted robustly and used as input for a rule-based action classifier.

The use-case in this paper focuses on lactating group-housed rabbits. Their agonistic interactions were quantified and used to test the automated pipeline in a commercial group-housing system. The study uses 20 pens, in each pen 4 unacquainted lactating does and their litters are mixed and group-housed for period of 10 days. Over time they developed social relationships, including dominance hierarchies. Studying these hierarchies and their dynamics is interesting from a fundamental animal behavior perspective, as this system could be used to test behavioral theories on the formation of social hierarchies among captive animals. Understanding the formation of social structures can be important for reducing aggression in group housing, with benefits for animal welfare and management. Although the social dynamics of group-housed does are not fully understood, a dominance hierarchy often appears to spontaneously emerge during the first days after the does and their litters enter the group pen^17^. This period is characterized by an increased frequency of agonistic interactions between the does (i.e., a general increase in the level of unrest in a pen), after which the frequency of interactions generally diminishes, indicating the establishment of a stable hierarchy^18^. Although these patterns are often observed and generally known, few ‘quantitative’ data or research methodologies are available for studying the dynamics of the frequency of these interactions (even at the pen level) and for testing hypotheses. Studies that rely on manual observation to measure the dominance hierarchy in rabbits mostly characterize the dyadic relationships using an ethogram. In this work, we primarily focus on the automated detection of the behaviors of chasing, fleeing and provocation of physical contact between does (possibly followed by a chasing/fleeing event) using video recordings. Agonistic interactions of this type are automatically detected using computer vision. Upon detection, the dominant and the subordinate animal within that interaction are automatically determined.

The main contribution of this work is the development and validation of a pure computer vision based pipeline for studying dominance hierarchies in group-housed animals. The problem was addressed in various stages. The first is tracking (and identification) of individual animals during highly active periods and extraction of movement patterns during these periods. Second, a rule-based system is presented that allows classifying the (dyadic) agonistic interactions between animals based on that movement pattern. By registering the temporal pattern of these dyadic interactions over a prolonged period of time, social dynamics can be monitored. Third, the inferred agonistic interactions (based on automated pipeline) are thoroughly compared and validated with observations made by a human observer, often considered a ‘silver standard’ in behavior research. Last, the detected interactions are used to visualize the evolution of the dominance hierarchy over time.

The remainder of this paper is organized as follows: results section shows experimental results, including an extensive validation of the procedure combined with discussion. Methods, introduces the pipeline used to automatically derive (an assessment of) the dominance hierarchy from raw video recordings. Within methods section, the data used to develop this pipeline is also described. Last, the most important insights and recommendations for future research are presented.

## Results and discussion

The following subsections present an extensive evaluation of the performance of the automated behavior detection pipeline and show how automated video analysis can be used to unravel social hierarchies.

### Group-level action segmentation

**Figure 1:**
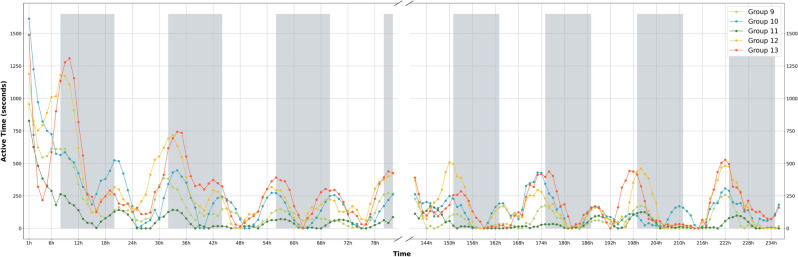
Visualization of the amount of activity at group-level as a function of time starting from the moment of grouping. The y-axis shows the Savitzky–Golay smoothed number of active seconds per hour, observed for five group pens obtained using the procedure described in the methods section. Shaded intervals refer to the light off times and negative values that arose as the result of smoothing procedure were ceiled to zero).

Figure 1 shows the evolution of the total activity (number of active seconds per hour) as a function of time for five groups obtained with the action detection procedure describe in Methods section. Without dilating on the (ethological) theories behind, this figure shows the generally known diurnal pattern of activity peaks around the transitions from light to darkness. Moreover, concurrent with this diurnal pattern is an overall trend of (exponentially) decreasing activity during the first few days after grouping. Also, some inter-group variability can be observed. Indeed, during peak activity when the lights are switched off, the active time of groups 12 and 13 is almost twice as large as the active time of the remaining groups during the first two days of the experiment, suggesting elevated levels of unrest in these groups. The increased levels of aggression after grouping^19^, the diurnal patterns^20^ and inter-group variability^21^ are known to occur in group-housed does and are a topic of research in particular in the context of animal welfare. Figure 1 illustrates that our processing pipeline can quantify these temporal patterns.

**Figure 2:**
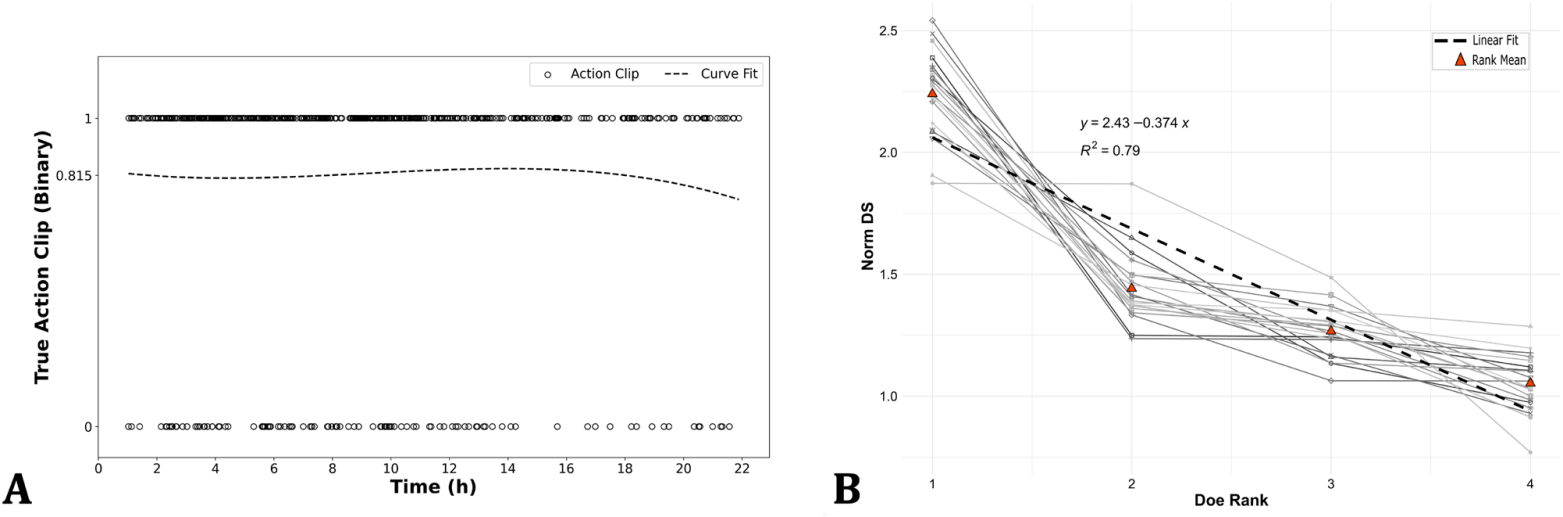
(**A**) Precision of the action segmentation as a function of time of day. Each marker represents one action clip and is assigned a value of 1 if at least one action was found in that clip by the human annotator and a value of 0 if not found. (**B**) Normalized David scores for each pen(lines)/animal(points), red markers point the mean normalized David score for the rank across all cages and the dashed black line represents the least squares regression line of rank averages.

It should be noted that this activity detector is sensitive to all types of movement and not only to activity related to agonistic behavior. Because it was developed as a temporal filter that would reduce the computational resources required for the following steps, it has a very high recall. No formal performance analysis was done to verify the recall but a general inspection suggests that no agonistic behavior involving significant movement is missed. To verify the precision, however, the 548 annotated clips for validation are used. Among those clips, 484 were found to include at least one example of agonistic behavior, resulting in an overall precision of 0.815. Also, when the timestamps of the action clips are used to plot the true (1) and false (0) positives as a function of the time of day, no time dependence of the precision was observed (Fig. 2A). This result indicates that the dynamics and trends of the agonistic actions involving chasing/fleeing behavior can be reliably assessed by inspecting the Savitzky-Golay smoothed plots. By multiplying them by a factor of 0.815, an estimate of the active time spent on agonistic interactions can be obtained.

### Doe detection and (re-)identification

Given a detected bounding box $\text{ bbox}_i$ and a ground-truth bounding box $\text{ bbox}_j$, the intersection over union (IOU) is defined as:

$$\begin{aligned}{} & {} \text {IOU}(\text {bbox}_i, \text {bbox}_j)&= \frac{\text {Area of Overlap} \, (\text {bbox}_i, \text {bbox}_j)}{\text {Area of Union} \, (\text {bbox}_i, \text {bbox}_j)}{} & {} \end{aligned}$$

When considering only bounding boxes with IOU of at least 0.5, the mean average precision (mAP) and recall were 0.924 and 0.733, respectively, after fine-tuning the detection model. Given the complex background, occlusions due to cage structure, and motion blur, the model accuracy was considered satisfactory and not optimized further. Moreover, the (re-)identification validation of the network achieved a test accuracy of 0.925 and loss of 0.1807.

### Quantification of agonistic interactions

Application of the procedures to video to select for action (segmentation), tracking and rule-based agonistic behavior detection acquired from all 20 groups resulted in the automated detection of 5480 agonistic interactions, where each detected interaction consists of: (1) the predicted time at which it was initiated, (2) the duration of the interaction, (3) the dominant animal, and (iv) the submissive animal according to that interaction. The results were compared with the expert-labeled described in Methods section below. Expert labels are only available for the first 24 h of 12 groups, so only the 1871 events detected in the first 24 h are used. Due to the discrepancies between the human annotator and the automated system, a one-to-one correspondence between expert annotations and automatically detected interactions cannot be easily obtained. Therefore, to check the correspondence between the automated and the manual procedure, precision and recall were computed using time windows (see Table 1). The recall for a $\delta t$ minute interval is computed as the ratio of the expert-observed interactions for which an interaction of the same type is observed at most $\delta t$ minutes sooner or later than the total number of expert observed interactions. This means that an observed interaction at time *t* where a first animal *A* dominates a second animal *B* (where the additional level of detail provided by the human-annotated who distinguishes between chasing, fleeing, etc. is not used) contributes to the numerator of the recall if in $t \pm \delta t$ the same interaction, i.e., *A* dominating *B*, is observed by the automated detector. The same principle applies to the precision, which expresses the ratio of the automatically detected interactions of a given type for which also an action of the same type was observed by the expert within a $\delta t$ minute interval to the total number of automatically detected interactions.

**Table 1:** Precision and recall (%) of automated behaviour detection algorithm compared against the annotations by animal experts.

Group #	Interval			
	1 Min		5 Min	
	Precision	Recall	Precision	Recall
1	0.5984	0.6869	0.6557	0.8086
8	0.6777	0.8209	0.8264	0.9269
9	0.6250	0.7577	0.7000	0.8782
10	0.6827	0.5892	0.8125	0.8468
12	0.7263	0.8019	0.7989	0.9189
17	0.6625	0.5912	0.7375	0.7390
18	0.7500	0.8507	0.8125	0.9030
19	0.6906	0.6221	0.7735	0.7689
20	0.6045	0.6439	0.8022	0.8744
21	0.8352	0.6667	0.8462	0.8009
22	0.7570	0.8382	0.8785	0.9129
23	0.5392	0.6667	0.6275	0.8402
**Mean**	0.6791	0.7113	0.7726	0.8516

The results in Table 1 demonstrate that the proposed pipeline on average has 0.68 precision and 0.71 recall in 1-min intervals. Within 5-min intervals, these values rise to 0.77 and 0.85, respectively. Specifically, it means that 77% of the automatically detected agonistic behaviors are also observed by the expert within a 5-min interval. Similarly, 85% of the annotated events by experts were recovered when searching in a time-range of 5 min around the automatically obtained detections. These results indicate a rather close correspondence between a human annotator and the automated interaction detection system. It should be noted that these tolerances are needed as a perfect temporal alignment between the human annotator and the automated system can not be expected. There are several reasons for that. For instance, the moment at which an aggressive interaction is initiated is often not unambiguously observable (it depends on the subjectivity of the human annotator and even when re-annotating for some interactions there can be quite some re-annotation variability especially when the level of action gradually builds up). Moreover, it is often debatable if an observed interaction is ‘new’ or rather the continuation of a previous interaction. Because of such reasons, one should consider the human annotations as a silver standard as opposed to a gold standard. It should be noted that extending the time window expands the search area for agonistic interactions identified by human experts, thereby increasing the likelihood of consensus between automated detections and expert annotations.

To gain insight into the type of errors made by the automated procedure, a random sample of 25 action clips was analyzed in detail. In this sample, 52 agonistic interactions were detected by the automated system. The trajectories that were found with the multiple object tracker were overlaid on the action clips to assess if the tracking algorithm and the succeeding interaction detector were indeed tracking the animals involved in the interaction and identifying them correctly. As a result, four detected interactions were found to be false positives in that sense that no agonistic interaction could be visually observed between the reported animals at that time and 1 id swap occurred where initiator and receiver were swapped. Last, three agonistic interactions could be visually observed that were not detected. Relatively spoken, 7.69% of the detected actions were false positives, 1.92% contains an id swap and 5.77% of the true agonistic interactions is missed.

**Figure 3:**
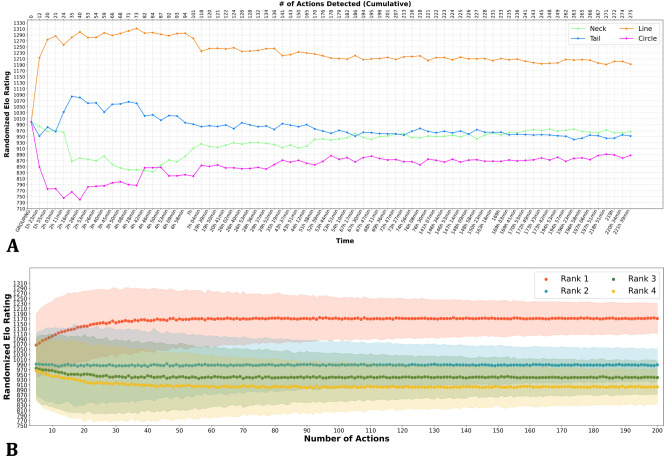
(**A**) Randomized Elo ratings of a group (#13) of 4 does (markings are indicated in the legend) over time representing the evolution of the hierarchy in a pen that is representative for this study. The randomized Elo ratings were computed using Equation 3 with 1000 permutations. (**B**) Simulation of the evolution of the expected randomized Elo ratings over time. The interaction probability matrix is used to simulate the rank scores (dotted lines: randomized Elo ratings) and the confidence intervals (shaded areas: 95% confidence intervals based on 1000 simulations). The rank refers to the rank of the animals in a hierarchy within their group.

### Social hierarchies and their dynamics

Figure 3A shows the evolution of the randomized Elo ratings as a function of time for one representative group (additional plots for other groups can be found in Appendix and relying on the automatically observed time dependent sociomatrix *S*(*t*). These plots show that rather quickly after grouping, one animal obtains a randomized Elo rating that is notably larger than the Elo ratings of the remaining animals, suggesting a hierarchical structure in which one animal dominates the remaining animals but without a distinct hierarchy among the remaining animals. Not dilating into ethological theories for this observation, this hierarchical structure is frequently observed among group-housed does^22^. The automated procedure successfully captures these structures and visualizes their dynamics. Moreover, validation with the human annotator revealed that the ranking of the dominant animals based on randomized Elo ratings was captured precisely, meaning that the dominant animal was always found correctly. Table 1 provides metrics at the behavior level on how well the automated pipeline performs. On the dominance level, randomized Elo ratings were computed using both the human observations (Elo ratings based on the human annotations were considered as the ground truth) and the automated pipeline. The resulting Elo ratings (4 ratings per cage) can be found in Appendix (Supplementary Table 1). The Pearson correlation coefficient between both types of Elo ratings was 0.901, showing a close resemblance between the human observer and the automated system.

In all 20 groups, it was observed that the Elo ratings of all does converge and that at equilibrium one doe had a notably larger Elo rating than the remaining three does. The dynamics were also very similar when considering the evolution of the Elo ratings as a function of the number of interactions. As a result, it is useful to consider the average evolution of the Elo ratings as a function of the number of interactions. This evolution is shown in Fig. 3B. To obtain this figure, we computed the $4 \times 4$ interaction probability matrix *P* in which the element $P_{i,j}$ is the probability that a randomly selected interaction (from all interactions that were automatically detected in the experiment) is an interaction in which the animal with rank *i* (where the rank is determined on the final randomize Elo ratings per group) dominates the animal with rank *j*. This matrix is:

$$\begin{aligned}{} & {} P&= \begin{pmatrix} 0 &{}\quad 0.1465 &{}\quad 0.1690 &{}\quad 0.1653 \\ 0.0557 &{}\quad 0 &{}\quad 0.0703 &{}\quad 0.0808 \\ 0.0505 &{}\quad 0.0571 &{}\quad 0 &{}\quad 0.0608 \\ 0.0467 &{}\quad 0.0476 &{}\quad 0.0496 &{}\quad 0 \\ \end{pmatrix}{} & {} \end{aligned}$$

By repeatedly sampling interactions according to the probabilities in *P*, the (expected) evolution of the randomized Elo ratings can be obtained (that provably converges to the average randomized Elo ratings at the end of the grouping experiment). Figure 3B was obtained based on 1000 repetitions and shows the average and 95% confidence intervals obtained using these simulations. This figure shows that the average randomized Elo ratings converge rather quickly but has a rather large uncertainty due to the of the interactions encoded in P. Identification of the dominant animal in the group with a high degree of certainty based on the randomized Elo ratings requires observation of a significant number of interactions. For instance, 64 observed interactions are needed to guarantee with a certainty of 97.1% that the dominant animal will be ranked first.

As a last illustration of how automated interaction detection can support the study of animal hierarchies, Fig. 2B shows the normalized David scores computed for each animal involved in this study based on all observed interactions, as well as the least squares regression line of the ranks on the normalized David scores. The vast amount of data generated by the automated interaction detection procedure enables study of not only estimates of the normalized David scores but also of the distribution of these scores and potentially also observation of deviant groups. For instance, in Fig. 2B, it can be seen that in one of the groups the normalized David scores of the first and second ranked animal is nearly the same, suggesting an unresolved competition for dominance. Closer inspection of the action clips, however, leads to the conclusion that one of the does found a strategy to escape its attacker by jumping onto a platform (a type of enrichment in the pens). As a result, the rule based system sometimes confused the dominant animal with the subordinate one.

### Practical use of the pipeline

It should be noted that the video data used in this study was originally collected for investigating the effect of group-housing on aggression, exclusively using human observation. Therefore, the original experiment focused on the broader context of assessing and improving animal welfare^23^ rather than the development of a computer vision pipeline. However, when using this pipeline on a routine basis, it can be optimized to reduce the computational demands and storage requirements. For instance, the group-level action segmentation step can be run on the fly to reduce storage requirements. Moreover, the analysis could be haltered after the hierarchy has been formed. However, these aspects were out of scope for this study. Also, a full analysis of the economical added value and its trade-off with animal welfare concerns is out of scope. However, in such applications the computational requirements are non-negligible. Therefore, we give an overview of the computation times and requirements in Appendix.

## Methods

### Ethics statement

All protocols and procedures were approved by the Ethics Committee for the Use of Animals in Research (EC 2021/389) of Flanders Research Institute for Agriculture, Fisheries and Food (ILVO). All international, national and/or institutional guidelines for care and use of animals were followed. The authors declare that the animal results of the study are reported in accordance with ARRIVE guidelines (https://arriveguidelines.org).

### Data

**Figure 4:**
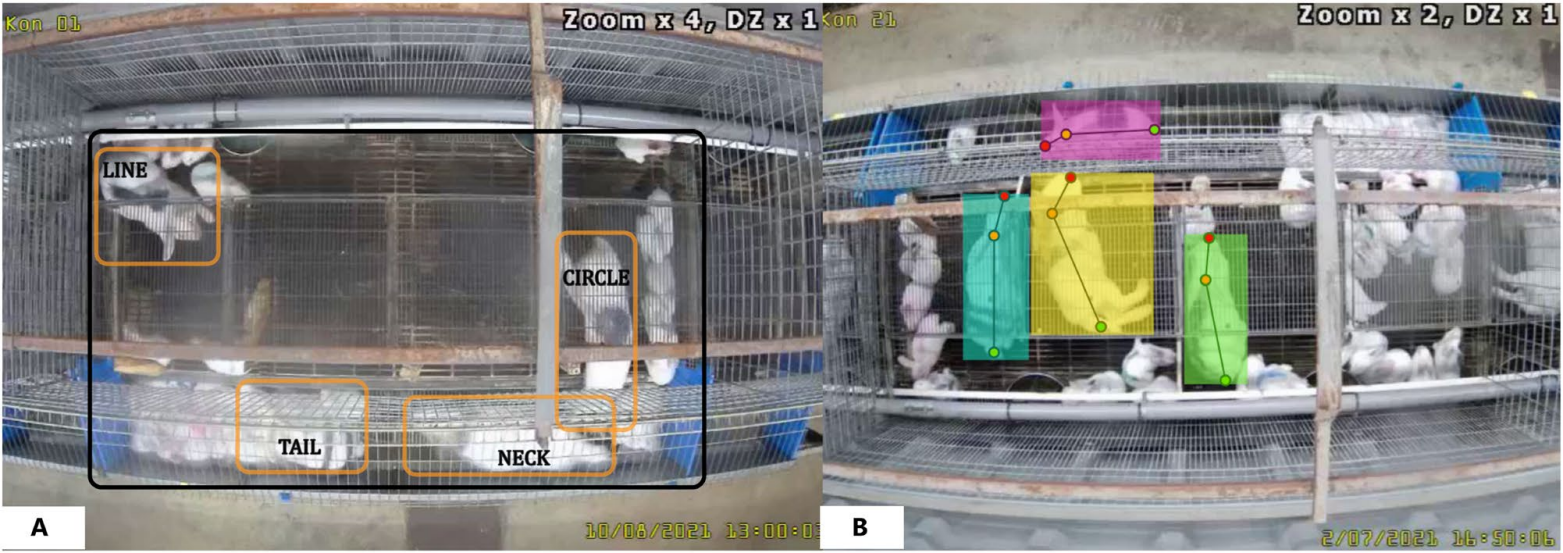
(**A**) A frame of four does and their litters housed in a group pen. Before processing, the area indicated by the black box is cropped from the video. The orange boxes highlight the locations of the four marked does in the cage. These markings (circle, line, tail and neck indicate the type of marker used identify the doe) were used for re-identification, (**B**) An example of an annotated frame. Each doe is delineated by a bounding box and three key-points (red: nose tip, orange: shoulder and green: tail implant) used for training the object detectors and pose estimation models.

The video recordings used in this work were made at a commercial rabbit farm in Flanders (Belgium) as part of a larger project investigating the effect of management practices on reproductive performance, welfare and behavior of group-housed farm rabbits^23^. Breeding does were first housed in single-litter cages open at the top with a surface area of about 50$\times$100 cm and an elevated platform of 50x30 cm. Four unacquainted does with their 22 day old kits switched from single-litter housing to group housing (achieved by removing the wire walls between four adjacent single-litter cages) for a total period of 10 days. Group pens were equipped with 2 feeders and 4 water nipples. A light schedule of 12L:12D was programmed. Does were marked with spray paint for visual identification; the same four markers were used in all group pens. Paint was reapplied when the markings started to fade (on average, one re-marking is applied per animal). The individuals were marked as ‘circle’, ‘line’, ‘tail’ and ‘neck’. A camera was mounted above the pen, offering a birds-eye view of the pen. The camera angle often included the side fences and the floor between the group pens but these were excluded from analysis. Cropping the image resulted in an exclusive focus on a single group pen (Fig. 4A).

Twenty nearly identical group pens were imaged in parallel from 10/08/2021 at noon (time of grouping) until 20/08/2021 in the morning. Only between 13/08/2021 at 23:00 and 16/08/2021 at 08:00 all cameras stopped recording due to a technical problem. Therefore, the total recording length is 8 days while the experiment spanned over 10 days. For group pen identification, the unique camera number (1-25) is used throughout the paper; 5 of the cameras were redundant as the experiment included 20 group pens. During the experiment, data on all group pens was collected repeatedly to check for skin injuries, weigh the rabbits and reapply individual markings. Under low-light conditions (night time for the animals) the camera switched to a night-vision mode using infrared (IR) illumination. The (re-)identification of individual animals during night-vision mode was impossible for a human observer due to glare. All actions detected by computer vision during the dark periods were therefore excluded from the analysis. Although group-level action segmentation and doe detection are possible in the dark, identification of the animals involved is not possible. Therefore, we only use daytime interactions and when we refer to interactions, we mean daytime interactions. Our analysis thus assumes that daytime interactions are representative for nighttime interactions. Additional details on the experimental design that are less relevant for the current work, yet recommended by the ARRIVE guidelines, are available in Appendix.

In total, 3520 h of uncorrupted video were recorded at a frame rate of 25 frames per second (fps). The video recordings were collected in MP4 file format using the H264-MPEG-4-AVC codec. The native resolution of videos before cropping (see Fig. 4A) was 720 $\times$ 480 pixels and all videos were saved on an hourly basis. Corrupted video files (empty or incomplete recordings) were eliminated from the analysis. Specifications of camera setup, storage facilities, computation time and hardware can be found in Appendix.

### A pipeline for agonistic action detection

**Figure 5:**
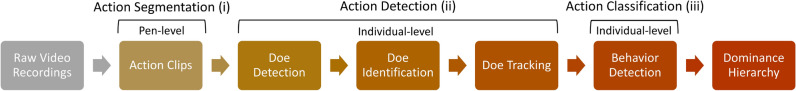
Visualization of the agonistic action detection pipeline (See Supplementary Figure 1 in Appendix for a more detailed algorithmic flow chart).

Rather than a single computer vision algorithm, the detection of agonistic behaviors and the identification of the animals involved in the interaction rely on an automated processing pipeline. This pipeline, visualized in Fig. 5 focuses on the detection of agonistic behaviors that involve a sudden movement of at least one of the does (chasing/fleeing or physical contact followed by fleeing) and consists of three main steps:

1. *Action segmentation at the pen level*. In this first step, a video is temporally segmented into action clips based on the presence/absence of movement of at least one doe. A new action clip starts when at least one doe starts moving and ends when all does return to a stationary state. The intermediate ’stationary’ clips are discarded. The length and frequency of these action clips as a function of time already supports a preliminary analysis of the dynamics of the agonistic interactions at the pen level.
2. *Action detection at the individual level*. The action clips are further processed separately. Per action clip, the does involved are tracked and identified (based on their marking). For each animal involved, its trajectory (the coordinates of its center of mass as a function of time) are extracted.
3. *Action classification*. The trajectories are processed by a number of hand-crafted rules to assess whether the movement patterns indicate that an agonistic interaction was present. If present, the dominant and submissive animals are identified.

The final result is a list of time-stamped agonistic interactions combined with an identification of which animal is behaving dominantly versus submissively within each interaction. This list is used as the basis for computing the dynamics of the dominance hierarchy within each group pen. The steps of this pipeline are detailed below and an algorithmic flow chart is available in the Appendix (Supplementary Figure 1). Details on the hardware specification, storage requirements and computation times for the steps in this pipeline can be found in Appendix.

### Action segmentation at the group level

The group pens allow the does and their kits to move freely within an area larger than single litter cages. During most hours of the experiment, does appear to remain stationary, punctuated by episodes of unrest which typically involve agonistic interactions such as aggression, chasing and fleeing. Those episodes usually involve significantly higher movement in the pens. As a result, video-based monitoring of the overall movement at the pen level makes it possible to discriminate between clips in which all does remain stationary and clips in which at least one doe moves intensively (‘action clips’). Only action clips are retained for analysis. Dense optical flow estimation was used to determine an action clip. Optical flow computation consists of finding the apparent motion of the objects in a scene as a result of the relative motion between the observer (top-view camera) and subjects (does). Assuming that the brightness of the moving objects does not change significantly in consecutive frames, dense optical flow finds a displacement field that maps one frame to a consecutive frame. By using the first frame of a consecutive pair of frames as a reference, each pixel is associated with a displacement vector consisting of the horizontal and vertical shift needed to map it to the corresponding pixel in the second frame of the pair. The method of Farnebäck^24^ was used for computing this displacement field. Developed with compensation for high-frequency vibrations in mind, this methods allows to compensate for the vibration due to shaking of the pens, which happens very frequently when animals (even in neighboring pens) bump into the side of the pen.

Prior to flow field estimation, the frame rate was downsampled to 1 frame per second and each frame was transformed to grayscale. Using the OpenCV (OpenCV-python v4.5.5.62) implementation of the Farnebäck method for dense optical flow, the displacement field was calculated with the following optimized parameter settings: pyramid scale = 0.5, number of levels = 3, window size = 15, number of iterations = 3, pixel neighborhood for polynomial expansion = 5, the standard deviation for polynomial expansion = 1.2^25^. Subsequently, the magnitude of the displacement vectors is computed as an estimator of the local motion. The average magnitude over the entire displacement field is used as a measure of total motion.

**Figure 6:**
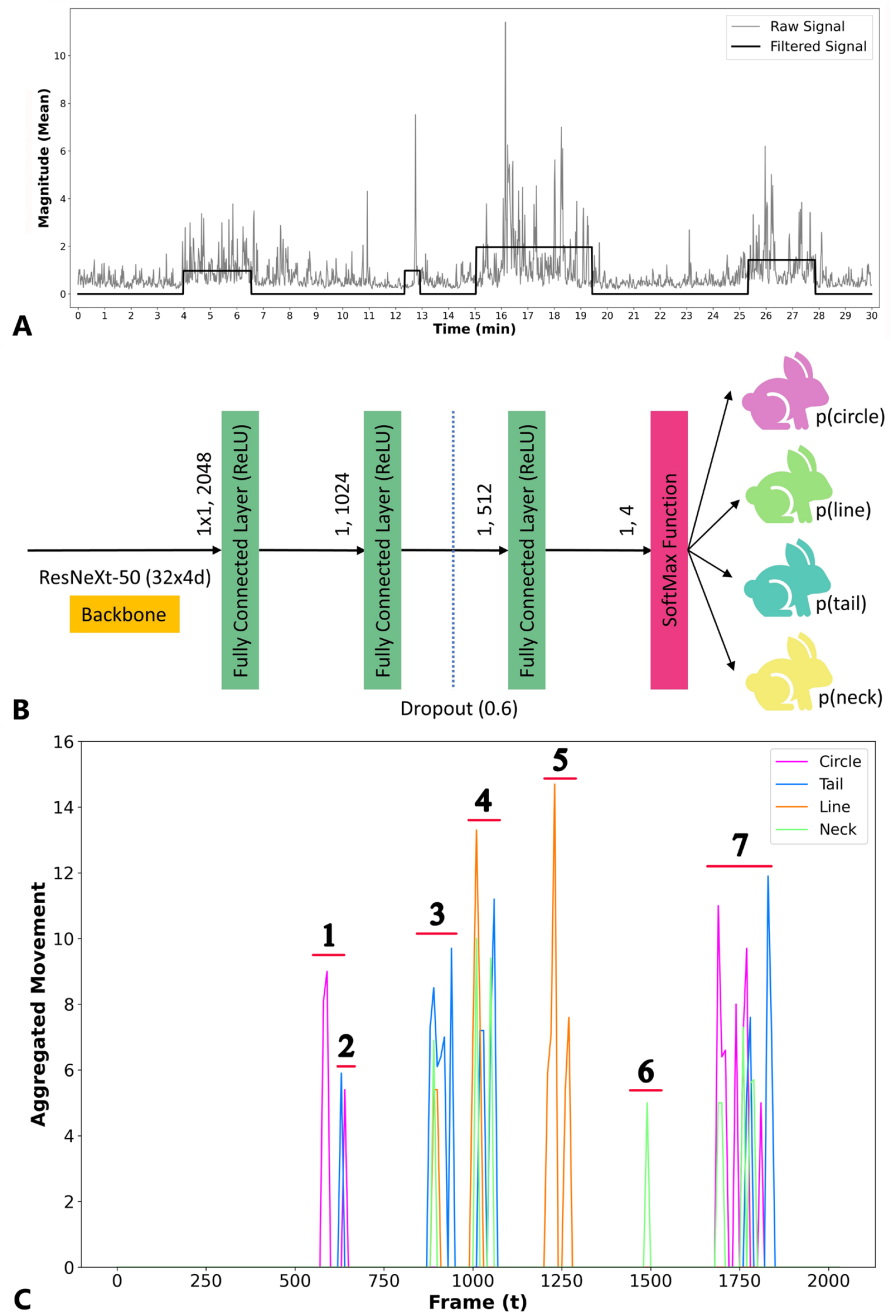
(**A**) Raw signal resulting from the optical flow algorithm and the filtered signal (used for segmenting the video) obtained after applying pen level action segmentation as described in Algorithm 1. The resulting intervals are used to characterize the group-level agonistic activity and are clipped for further analysis (Group 1, Day 1, 18 h), (**B**) Visualization of the customized neural network architecture consisting of a ResNeXt backbone followed by three fully connected layers (numbers indicate the hidden size of each layer) with ReLu activation and a dropout layer with a 0.6 dropout rate between the second and third fully connected layer. A softmax layer is used to obtain the final probabilities. (**C**) Evolution of the (filtered) animal-level velocity (expressed as the number of pixels per frame) during one action clip of one group. The numbers (and horizontal lines) indicate movements that are considered as event groups.

A univariate time series of the magnitude of movement in a cage as a function of time is obtained by applying the process described above to the video recordings of that pen. The time series starts at grouping and ends after 10 days of group housing with a resolution of 1 measurement per second. This time series is post-processed to distinguish between fast (running) movement of at least one of the does (indicative of an agonistic action) and more subtle movements by the does or the kits or very brief bursts of movement that are not related to agonistic behavior (both are considered as noise in the time series). Moreover, agonistic behaviors sometimes contain short (< 60 seconds) gaps between actions, which can be either a continuation of the event or discrete events following each other. The most important components of the post-processing are described below; for a complete description see Appendix. Noise is removed by suppressing all magnitudes below 1 (meaning that all frames where the average displacement is smaller than 1 pixel are set to zero). Subsequently, a rolling median filter with a window size of 7 seconds is used, followed by a mean filter with a window size of 15 seconds to smoothen the signal. To compensate the smoothing of the edges at the start and end of an action moment, a procedure similar to a 1D dilation filter is applied. To fill temporary drops in the magnitude (due to short interruptions) high-magnitude intervals less than 60 seconds apart are merged by replacing the magnitudes in between with the median of the neighboring intervals. The average intensity of the resulting intervals was finally used to determine if it is retained as an action interval (average magnitude >1.2). The result of this process is a binary time series of action intervals and corresponding action clips. A sample is shown in Fig. 6A. This procedure is applied to all 20 group pens. The obtained action clips (and their timestamps) are used in the next step of the pipeline.

To visualize the long-term trends in the frequency of action, the binary time series is further aggregated at the hourly level by computing the total number of seconds of action per hour. To improve interpretability, the resulting signal was smoothed using a Savitzky-Golay filter^26^ implemented in SciPy (v1.7.3)^27^ with window size of 11 and polynomial order of 3 before plotting.

### Action detection: doe (keypoint) detection

Further processing of the action clips is needed to automatically identify the dominant and submissive animal according to the behavior observed in each clip. Such identification was based on the movement patterns of the animals involved. To extract these movement patterns, does are detected, (re-)identified and tracked throughout an action clip. In this section, the detection procedure is described; the (re-)identification and tracking procedures are described in the sections that follow.

The presence of dozens of kits, variations in lighting conditions and camera focus, and small variations in cage architecture all complicate the development of a robust detector for does. As deep learning based object detectors excel in accuracy and robustness, especially in complex environments, we chose them for this study. To train the object detector, 300 frames were selected randomly from all videos. Additionally, 167 more frames were hand-picked from the action clips (during light hours) and added to the data, resulting in 467 training images containing 1695 detectable does (with 4 does present in each frame, this means that 90.7% of the does are detectable by the human annotator). Additionally, 25 frames (with 95 human-detectable does) selected for model validation/tuning and 25 with 91 human-detectable does for testing. All images were annotated using COCO Annotator (v0.11.1)^28^. For each doe, a bounding box and three keypoints were annotated (tip of the nose, shoulder and base of the tail). An example of an annotated frame is presented in Fig. 4B. The Decectron 2 (v0.6)^29^ together with pretrained Keypoint R-CNN network X-101-32x8d-FPN was used. This particular network uses Mask R-CNN and a Feature Pyramid Network in combination with ResNetXt (101 layers) as a backbone^30,31^. Although the pretraining of the keypoint detector is mainly done using a dataset of images of humans with keypoint annotations, our results show that this network can easily be fine-tuned for our use case via transfer learning. During fine-tuning, the base learning rate is set to $5e^{-4}$ and *WarmupCosineLR* is used as the learning rate scheduler. The maximum iterations parameter is set to 5000.

During the agonistic behaviors of chasing and fleeing, the presence of motion blur and the stretching of the animals complicates the detection. However, detection of agonistic behaviors is based on reliable detection of the animals in these moments. For this reason, both training and test sets were constructed to contain moving animals. Despite the high precision of the keypoints in general, the keypoints are found to be unstable when quick movements occur. We observed that the mean average precision (mAP) on the key points^32^ was only 0.8175 during the interactions (validation set of 20 does) compared to an overall mAP of 0.9701 (validation set of 95 does). The fine-tuned object detector is used to process each action clip (at the original frame rate of 25fps). In total, over 10 million frames were analyzed. Predicted bounding box and keypoint coordinates are stored in JSON files. For each predicted bounding box, the confidence put out by the neural network is stored as well.

### Action detection: doe (re-)identification

Using the markings described in data subsection, the does were identifiable. Markings were quite variable between pens and also varied over time due to paint wearing off and being reapplied. Therefore, (re-)identification of the detected rabbits is generalizable only if the identification model can embrace these variations. For this a deep learning-based multi-class classification model was used. To collect training examples of each marking, the bounding boxes predicted by the doe detector were used to crop the does from the frames. In total, 1560 cropped does were selected and assigned to one of four classes based on their markings. Train and test data were randomly selected using a 9/1 split.

The crops were resized to (224, 224) pixels and pixel intensities were normalized using the mean and standard deviation calculated from the ImageNet dataset^33^. Random flipping was used as a data augmentation procedure. An implementation of ReSNeXt model with 50 layers, 32 grouped convolutions and 4 dimensions per group^34,35^ in the PyTorch framework was used as an architecture. Pretrained weights (after pretraining on the ImageNet dataset) were fine-tuned using the training data. Moreover, the fully connected layer of the ReSNeXt model is replaced with the sequential architecture shown in Fig. 6B, which gradually reduces the dimensionality of the feature space. In the last layer, a softmax function is applied to get probabilities of identification.

The categorical cross-entropy loss is used as a loss function, which is minimized using the Adam optimizer. In a careful hyperparameter optimization process, the optimal learning rate was found to be $1e^{-4}$. The model trained 50 epochs and the restored checkpoint with the lowest validation loss was used as a final model. The fine-tuned model was applied to each detected doe using the methodology in the previous section. For each detected doe, this model outputs a probability estimate for each of the four markings. These estimated probabilities are appended to the JSON file containing the location of the bounding boxes and the keypoints and are used for further processing.

### Action detection: tracking caged animals

To analyze the movement patterns, the animals need to be tracked over time. In the ideal case where the doe detector and the identification model are flawless, tracking is trivial. However, an analysis of the performance of these models reveals that tracking is not so simple. Mostly due to partial occlusion (e.g., by a fence or another animal) or the posture of the animals, markings are not visible in some frames. Moreover, the detector sometimes confuses a group of tightly packed kits for a doe or misses a doe when surrounded by many kits. Even for a human observer, locating the four does proves to be challenging in some frames. As a result, more sophisticated multiple-object tracking (MOT) algorithms need to be used. Popular examples of such trackers are SORT, which is also used in DeepLabCut^36^ for tracking animals in video, or trackers that compute similarities between detected objects to associate bounding boxes, a principle that is also used in SLEAP^37^ for multiple-animal pose tracking. Despite their popularity, the results obtained with initial experiments with these trackers were not satisfactory, mostly due to the following reasons: (1) the doe detectors are more error-prone than the applications in which these trackers are traditionally used; (2) doe movements change abruptly and, most importantly, (3) these general-purpose trackers do not take into account that the same (in our case four) animals are always present in each frame. For example, consider a setting in which three out of four animals can be identified with high reliability. Although the identity of the remaining animal can be easily derived, the general purpose trackers do not exploit that logic because they were developed for a scenario where objects can enter and leave the scene.

As a result, a dedicated multiple-object tracker was implemented. The high-level structure of this tracker is rather simple (see below for description). For more details, we refer to our extensively documented GitHub repository (https://github.com/nusretipek/hierarchy_of_rabbits). Our MOT consists of 7 steps that are executed sequentially: (1) Four empty tracks (one for each doe, i.e., circle, tail, line, and neck) are initialized and all bounding boxes in which the animal can be identified based on its marking with a confidence of at least 0.98 are added to the corresponding track. (2) A spatio-temporal filter is applied to the (partial) tracks from the former step, which removes temporally or spatially isolated bounding boxes (due to the frame rate of 25fps, it assumed that a correctly identified doe in a given frame should also be identifiable in the preceding and following two frames at a nearby position which high confidence). Bounding boxes that do not meet these criteria are removed from the tracks. (3) In frames in which three out of four does are identifiable, the remaining bounding box (if present) is automatically assigned to the remaining track. Combining the former steps already assigns about half of the bounding boxes to a track. We refer to these boxes as anchor boxes. (4) Gaps (a sequence of frames in which no high-confidence bounding box for a track is found) in the tracks are filled by greedily linking the bounding boxes in the frames that constitute the gap using nearest centroid linking. This generates four candidate tracklets to complete the track of the doe under consideration. The average confidence of each candidate tracklet (arithmetic mean of the confidences for that doe of the constituting bounding boxes) is computed. If the average confidence of one of the candidate tracklets is significantly larger than that of the remaining candidates, that tracklet is used to complete the gap. (5) In the (very rare) case that a bounding box is assigned to more than one track, it is removed from all tracks but the one with the largest confidence. (6) Gaps that still remain are filled by linearly interpolating the centroids of the anchor boxes at the beginning and end of the gap and the interpolated values are flagged as being computer-generated and this less reliable. (7) The sequence of centroids that constitute a track are spatiotemporally smoothed (spatiotemorally outlying centroids are removed and replaced with linear interpolation, and a rolling average filter is used to account for positional variation due to small positional errors of the bounding boxes).

The tracking procedure is applied to each action clip and the centroids are stored for further processing.

### Action classification: automatic behavior detection

**Figure 7:**
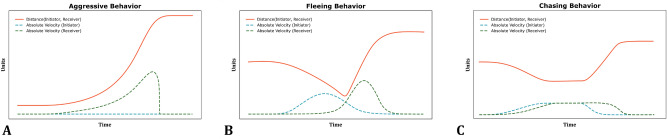
Conceptual plots on how distances and absolute velocities between interacting does vary in function of time for three types of idealized agonistic interactions.

To detect the presence of agonistic behavior in the set of tracks derived from the action clips, one of the following approaches can be taken. First, a data-driven approach involves machine learning algorithms that learn how to map the tracks to a behavior by using an extensive amount of expert-labeled data. A second approach relies on hand-crafted rules to classify the tracks. Despite the potential of the former approach, sufficient expert-labeled data was not available for use here. The rule-based approach was therefore used to build an action classifier. As a starting point, we considered interaction types between a pair of does labeled as agonistic: (1) aggressive behavior, (2) fleeing behavior and (3) chasing behavior. These three agonistic interaction types can be schematically described fairly easily by considering the absolute velocities of the two animals involved and the distance between them as a function of time. Figure 7 shows the evolution of absolute velocity of initiator and receiver together with distance between them as a function of time in an idealized settings. In Fig. 7A, animals are initially stationary and in close proximity. A trigger such as a biting event causes the other animal to flee, thus increasing velocity of the receiver and its distance to the initiator. Fleeing behavior is an example where the initiating doe approaches the other doe (reducing the distance) and when the animals are in close proximity, the initiator becomes stationary and the receiver moves away from the initiator (Fig. 7B). Finally, chasing behavior is characterized by rather constant, but elevated velocities of two animals in close proximity for some time after which the distance increases again (Fig. 7C). These patterns form the basis for a set of rules that allow detection of agonistic interactions involving a high level of activity, i.e., chasing, fleeing and provocative physical contact between does. Importantly, less explosive agonistic interactions such as threatening, slowly approaching, freezing and other sub-categories of agonistic behaviors are not considered in this paper. An example of such a rule from the observed trajectories *A* and *B* of two rabbits is (see GitHub repository for the complete list);

$$\begin{aligned} \begin{aligned} {\textbf {If}} \,\,&\text{ Chebyshev } \text{ distance }(A, B)_{t=t_0} \le 50 {\textbf {\,and}} \\ {}&\text{ absolute } \text{ velocity }(A)_{t = t_0-10,\ldots , t_0} \le 5 {\textbf {\,and}} \\&\text{ absolute } \text{ velocity }(B)_{t = t_0, \ldots , t_0+10} \ge 5 \; \\ {\textbf {Then}} \\&\text{ Agonistic } \text{ action }(\text{ Initiator }=A, \, \text{ Receiver }=B) \end{aligned} \end{aligned}$$

It is important to note that agonistic interactions tend to be temporally clustered. For instance, when the initiator animal bites a receiving doe that subsequently flees, it is not uncommon that within a short time-span a similar interaction, for instance a chasing interaction, occurs. In this case, the latter action should be considered as a continuation of the former, rather than a separate action; this is known as a ‘behavior bout’. Additionally, a continuous action such as chasing or multiple aggressive behaviors in a short interval create risks for duplicate classification. Moreover, the typical circling behavior of does is enhanced in a confined environment. To address these issues, we temporally cluster all the observed movements. More precisely, action clips are segmented based on the presence/absence of movement of at least one animal. Consecutive frames in which at least one animal is moving, ultimately form one segment (see Fig. 6C for an illustration).

One event group is the union of the generated tracks of the animals that move within one segment. Within one event group at most one interaction between each pair of animals is reported (assuming that other interactions are merely repetitions or a continuation of the same action) and it is assumed that no cycles occur where, for instance, the doe with the circle marking chases the doe with a tail marking which then again chases the doe with a circle marking. This happens when one animal initiates an action with a second one and, due to that action, a third animal starts moving as well. In this case, we broke the cycles by removing the interactions chronologically backwards in time until there are no cycles, forming a directed acyclic graph (DAG).

The above rule-based system was applied to all trajectories, resulting in a series of time-stamped interactions per cage. The obtained interactions are converted into a *time-dependent sociomatrix* *S*(*t*):

1 $$\begin{aligned}{} & {} S(t)&= \begin{pmatrix} s_{1,1}(t) &{}\quad \cdots &{}\quad s_{1,4}(t) \\ \vdots &{}\quad \ddots &{}\quad \vdots \\ s_{4,1}(t) &{}\quad \cdots &{}\quad s_{4,4}(t) \end{pmatrix} \,,&\quad&\end{aligned}$$

where for any *i*, *j* element of $\{1,\ldots ,4\}$, $s_{i,j}(t)$ represents the number of detected interactions between animals *i* and *j* with a time-stamp smaller than time *t*; and in which the $i^{th}$ animal dominated the $j^{th}$ animal.

### Analysis of dominance hierarchies

Several methods for hierarchy quantification can be found in literature. A dyadic agonistic interaction matrix, which can be created automatically by our pipeline, is often the basis for such an analysis. Prominent examples include the direction consistency index (DCI), Landau’s h and its improved version ($h'$) which are often used as linearity indices, circular triads and steepness and (randomized) Elo ratings^38–41^. The values for these indices can be derived from sociomatrices. Importantly, as our automated pipeline is capable of producing an accurate time-dependent variant *S*(*t*) of this sociomatrix, the derived indices also become time-dependent and therefore allow for study of the dynamics of hierarchy formation. In this work, we use the randomized Elo ratings^41^ per animal to quantify the position of an animal in the hierarchy. The randomized Elo rating at time *t* for each animal can be derived from *S*(*t*) as follows: (1) let *n*(*t*) be the total number of interactions in *S*(*t*); (2) generate a random sequence of *n*(*t*) interactions such that the number of times that the $i^{th}$ animal dominates the $j^{th}$ animal is equal to $s_{i,j}(t)$; (3) Set the initial Elo ratings $R_1, \ldots , R_4$ of the four animals equal to 1000; (4) iterate through the generated sequence of interactions and for each interaction do the following (let *i* and *j* be the animals involved in the current iteration and let *i* be the dominant animals according to this interaction): compute

2 $$\begin{aligned}{} & {} E_i&= \frac{1}{1 + 10^{(R_j-R_i)/400}}{} & {} \end{aligned}$$

update $$R_i$$ and $$R_j$$

3 $$\begin{aligned}{} & {} R_i&\leftarrow R_i + 100(1-E_i)\,, \qquad R_j \leftarrow R_j - 100 E_i\,,{} & {} \end{aligned}$$

and store the final value; (5) repeat steps 2–4 a large number of times (in this paper 1000 repetitions were used); and (6) compute the average of all repetitions.

By applying this procedure to each *S*(*t*), a time-dependent randomized Elo rating is obtained. This scheme is applied to compute the time-dependent randomized Elo rating for each group. To improve the reproducibility of this work, this procedure was implemented in a (new) Python package HierarchiaPy (https://pypi.org/project/HierarchiaPy) along with several other popular dominance metrics.

### Expert annotations

To validate the accuracy and precision of the automated behavior detection pipeline, a subset of the video monitoring data were annotated by a human annotator. To obtain these annotations, 548 action clips were extracted by applying the cage-level optical flow-based action segmentor to the first 24h of video of each of 12 groups. A trained annotator then annotated them using the Noldus Observer XT14^42^. Agonistic interactions were scored continuously for all does in the group pens. The used ethogram contained the following agonistic behaviors: attacking (quick movement towards another doe, neck stretched out, ears flattened, physical contact is made), threatening (quick movement towards another doe, ears flattened but no physical contact is made), fighting (two does get into a fight by gripping each other with their teeth and/or scratching with the hind claws), circling (two does are locked by gripping each other with their teeth, a circular movement may occur), counter attack (doe reacts by attacking a doe that first attacked), fleeing, and chasing. For each behavior, the initiating and receiving doe were registered. In total, 6,344 agonistic interactions (divided over 12 groups) were observed by the annotator, allowing a rigorous comparison. It is important to note here that that the degree of detail of the expert annotations differs from that of the automated system. Regarding model validation, two aspects are particularly important. (1) Attacking/threatening behavior is often followed by fleeing of the submissive doe. The human annotator notes these as two consecutive events whereas the rule based system recognizes this as one event in which the attacker is classified as dominant in relation to the fleeing animal. (2) The human annotator often indicates short interruptions in an attacking, chasing or fleeing behavior by registering several events instead of one, whereas the rule-based system recognizes this as one behavior. Even though a formal analysis is not available, merging such behaviors could profoundly reduce the number of detected behaviors.

The former annotation campaign generated data that can be used to validate the automated pipeline. Moreover, to gain insight into the modes of failure of our pipeline, for a random selection of 25 action clips, a human annotator additionally cross-matched the detected actions using the pipeline with the raw video material. Three types of potential mistakes were distinguished: (1) false negatives, meaning that a true agonistic interaction was not detected; (2) false positives, meaning that a non-active moment was falsely identified as an agonistic interaction; (3) id-swaps, meaning than an action was detected but the animals involved were misidentified.

## Conclusion

Computer vision based automatic monitoring technologies provide opportunities to study social interactions between animals in real-life settings. The availability of continuous recording data makes it possible to automatically recognize a set of agonistic behaviors among identified animals. We present and validate an agonistic interaction detection pipeline that enables study of hierarchy formation. Most computer vision applications that support study animal behavior focus on individual behaviors or rely on artificial experimental setups involving only a limited number of animals under laboratory conditions. The present study shows for the first time that this technology can also be used to study interactions in complex environments. Dominance analysis is based on a large number of studies of dyadic interaction data, but fewer studies address the temporal aspect (evolution of the hierarchy). The proposed automated behavior identification pipeline not only complements a labor-intensive and subjective process of annotation and makes it easy to obtain temporal insights. One limitation of the current approach is that it involves a significant amount of engineering to construct and fine-tune the entire pipeline. Moreover, due to its heavy reliance on movement patterns, more subtle signs of agonistic behavior cannot be identified by this approach.

### Supplementary Information

Supplementary Information.

## Data Availability

A sample of raw videos, data generated and/or analysed during the current study are available in the Agonistic Rabbit Action Detection/Classification repository, https://zenodo.org/record/7248029 (DOI: 10.5281/zenodo.7248029). Specifically, we provide continuous 6 h of raw video from Group 1, segmented action clips from those 6 h, object detector and (re-)identification network model weights, COCO-format annotated bounding box and keypoints (with their images) and automatically detected agonistic actions from Group 1 (6 h). Full dataset of raw videos analysed during the current study are not publicly available due large amount of data but are available from the corresponding author on reasonable request.
