# Supplementary material for: Quantifying agonistic interactions between group-housed animals to derive social hierarchies using computer vision: a case study with commercially group-housed rabbits
Source: Sci Rep. 2023 Aug 29;13:14138. doi: 10.1038/s41598-023-41104-6 (PMC10465565; doi:10.1038/s41598-023-41104-6)
Supplement: Supplementary file 1 — Supplementary Information. [file 41598_2023_41104_MOESM1_ESM.pdf]

Appendix

Animals and experimental design, following ARRIVE guidelines

This Appendix contains experimental details that are less relevant for the current work (and therefore are not reported in the main text) but guarantee compliance with the ARRIVE guidelines.

Study Design

A total of 105 breeding Hyla does (Sevremoine, France and Valli del Pasubio, Italy) were randomly selected from a commercial rabbit farm in Flanders (Belgium). Does were selected from the present batch of breeding does between the 3rd and 5th parturition. Does were housed on a farm in single-litter cages of 100 x 50 cm with an elevated platform of 50 x 30 cm, open roof, plastic slatted floor and wooden gnawing block. Animals were, according to common farm practice, subjected to a reproduction round of 42 days. In each round, does were artificially inseminated (AI) 10 days after giving birth to their litter. One day post-partum (pp) live-born kits were cross-fostered at 11 or 12 kits per doe (the surplus of kits were euthanized). 32 days pp does and their kits were moved to a different cleaned compartment and were housed in groups of four does with their kits in enriched group pens. After three days, 35 days pp, does (by then already advanced 25 days in their next pregnancy) were removed back to single-litter cages to prepare for their next litter which was expected seven days later, resulting in a reproduction round of 42 days. Does were provided with a nest box and nesting material (flax and wood shavings) three days prior to parturition. Kits remained in the multiple-litter group pens until slaughter age (10-11 weeks). All animals were given commercial pellet rabbit feed (Quartes NV (Deinze, Belgium)) and water ad libitum and had access to a wooden gnawing block. A light round of 12L:12D was programmed, except 7 days prior to AI when the light round was changed to 16L:8D. Light intensity was higher than 40 lux during the light hours. The air temperature was set at 20-21 °C and relative air humidity was between 60% and 75%.

Sample Size, Inclusion Criteria and Exclusion Criteria

From the farm, 105 multiparous does (between the 3rd and 5th parturition) were selected for the trial and housed in single-litter cages. From these selected does, 80 does and their litters were housed in groups 22 days pp. This deviated from the standard management practice of the farm where group housing was only realized 32 days pp. Instead of moving does and their kits to group pens, mobile wire walls between four adjacent single-litter cages were removed to create a larger group pen. Group pens were assigned one of the following treatments in a completely randomized block design (n = 3 per treatment, figure 1): provision of small pressed alfalfa blocks as distraction material (A), three wooden panels attached underneath the platforms, visually separating the pen into four areas (P), both alfalfa and wooden panels (AP), or no extra enrichment (controls, C). Group housing lasted for 10 days, ending at 32 days post-partum, after which does and their kits were moved to another compartment as described above. This experiment was replicated for three consecutive reproduction rounds (June - October), resulting in a total sample size of 12 group pens per treatment. Between rounds, does were assigned to a different group-pen to account for possible location/pen effects, to a different treatment and three unfamiliar does. Does that were selected for the trial but were not housed in the group (n = 25) were kept as spare does in single-litter cages to replace non-pregnant, sick, injured or deceased does in between reproduction rounds but not during the group housing phase. Prior to the experiment, all animals were in good body condition and showed no signs of sickness.

Outcome Measures, Statistical Methods, Experimental Animals and Procedures

After the collection of video footage from all pens using a top-view camera, 12 randomly selected pens (3 in each treatment) agonistic interaction annotated by an animal expert using Noldus Observer XT14. The outcome of the technology development is to robustly assess the hierarchy formation after group housing using computer vision. Hence, dominance analysis metrics, specifically, randomized ELO ratings are used as a measure of dominance within the pens. There are no experimental procedures applied to the animals such as pharmacological, surgical or pathogen injection. Therefore, the stress of the animals is limited to only renewing the marking of the animals every 48-72 hours during the video footage collection stage.

Hardware specification, storage requirements and computation time

The cameras used for recording are Avtech AVM543p and AVM5447P cameras. A workstation (PC HP Z2 G4 with Intel Core i7 8700 CPU and 16 GB RAM) was used to capture the recordings in a local SSD disk (1 x 4 TB). The cameras are powered using a Power over Ethernet (PoE) switch, Zyxel 1920 24HPV2. Blue Iris V5 software was usedfor recording.

For processing, the videos were uploaded to the VSC (Flemish Supercomputer Center) data server for action segmentation. Since the videos are already saved using the H.264 video compression standard, no further compression was applied. After the action segmentation, the action clips are stored on the data server ($\approx$78 GB) for further processing.

Action segmentation was performed using AMD EPYC 7552 processors (AMD Zen2 micro-architecture, a.k.a. AMD Rome) chip. During processing, at most 2.6 GB RAM is used. Processing one hour of raw video has an average CPU-time of 5 minutes and 22 seconds, including file I/O and group-level action segmentation (single core).

The later stages of the pipeline are exclusively computed on the daytime action clips detected in the first step (about 106 hours of action clips). In other words, 1 hour of raw video can contain 0 (no further processing) to 3600 seconds (process entire hour) of action. We report the processing time for needed for processing 1 hour of action clips for consistency. The doe detection and (re-)identification is computed using a single NVIDIA GeForce RTX 2080 Ti GPU coupled with Intel(R) Xeon(R) Silver 4110 CPU and 128 GB Ram. The training time for the doe detection network was 34 minutes and 59 seconds. The training time for the doe (re-)identification network was 12 minutes and 20 seconds. The inference time for 1 hour of action clips is approximately 180 minutes for detection and 78 minutes for identification (using 4 parallel processes in a single GPU). Both training and inference are done using GPU.

Algorithmic flow chart

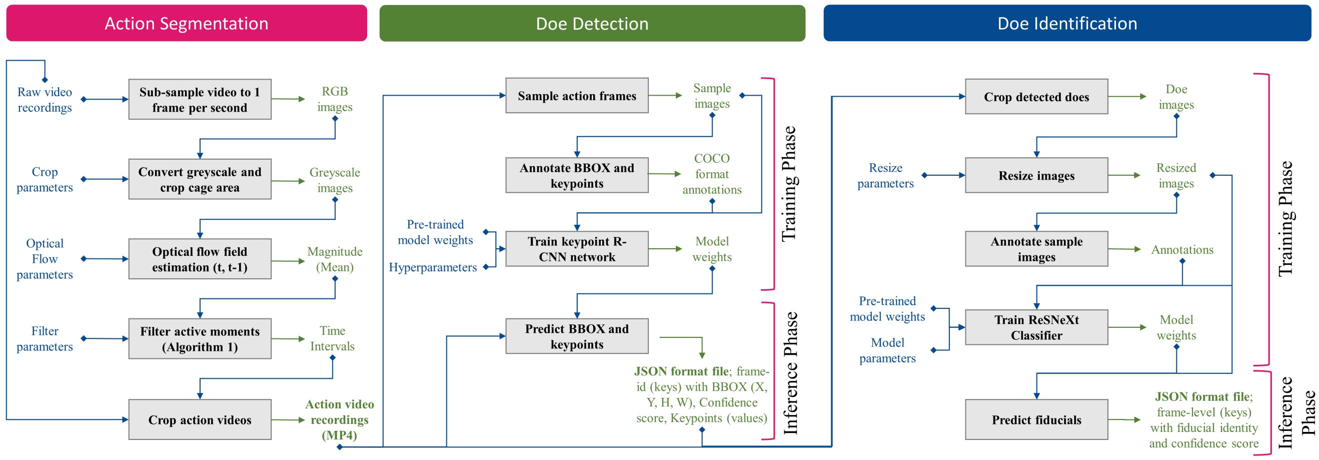

Supplementary Figure 1. Algorithmic flow chart for individual components of action segmentation, doe detection and doe identification. The inputs are shown in blue, the outputs are shown in green.

Dominance-level comparison: Randomized Elo ratings

Supplementary Table 1. Randomized Elo ratings for human annotations and automated annotations, both the starting value and the number of random permutations were set to 1000.

Group #	Annotation Type							
	**Human**				**Automated**			
	Circle	Tail	Line	Neck	Circle	Tail	Line	Neck
1	1273	973	872	883	1203	934	918	945
8	975	890	1278	857	1016	907	1185	892
9	1242	934	959	865	1221	856	919	1004
10	788	979	1356	877	927	983	1247	842
12	1265	990	902	843	1138	935	1012	914
17	1218	947	941	894	1129	889	976	1007
18	865	1306	894	934	947	1222	923	909
19	1008	1200	885	907	899	1191	971	940
20	901	923	996	1180	946	907	955	1192
21	1219	949	914	919	1206	931	842	1021
22	1107	990	899	1003	1081	1077	834	1008
23	930	885	920	1265	1004	945	937	1114

Algorithm of action clip filtration

Algorithm 1 Filtering Algorithm of Mean Magnitudes

```
procedure WHITE_NOISE_REMOVAL(winSize)
    for each actionClip do
        [actionClip ≤ 1] ← 0
        for j ∈ (winSize, len(actionClip) − winSize) do
            low_i ← j − winSize
            high_i ← j + winSize + 1
            nZero ← len(actionClip[low_i, high_i] == 0)
            if nZero/(2 ∗ winSize + 1) ≥ 0.5 then
                actionClip[j] ← 0
            end if
        end for
        for j ∈ (0, length(actionClip) + 15 + 1) do
            actionClip[j] ← mean(actionClip[j, j + 15])
        end for
        [actionClip ≤ 0.5] ← 0
    end for
end procedure

procedure EXPAND_INTERVALS(expSize, operation)
    for each actionClip do
        prev ← 0
        next ← 0
        for i ∈ (expSize, len(actionClip) − expSize) do
            if prev + next + expSize > i then
                Continue
            end if
            if arr[i] > 0 then
                next ← 0
                j ← length(actionClip) − expSize
                Cond_i ← j > next + i
                Cond_j ← actionClip[i + next] ≠ 0
                while Cond_i & Cond_j do
                    next ← next + 1
                    if operation is max then
                        v ← max(actionClip[i, i + next])
                    else if operation is mean then
                        v ← mean(actionClip[i, i + next])
                    end if
                    low_i ← i − expSize
                    high_i ← i + next + expSize
                    actionClip[low_i : high_i] ← v
                    prev ← i
                end while
            end if
        end for
    end for
end procedure
```

Algorithm of filling in-between anchor points

Algorithm 2 Filling in-between Anchor Points

```
for each track do
    for each interval([p_1:p_n]) do
        dist ← euclidean_dist(p_0, p_{n+1})
        candidateTrack ← getCandidatetrack(p_0, p_{n+1})
        if candidateTrack is None then
            eTrack ← linear_extrapolation(p_0, p_{n+1})
            ca ← mean(confidence(p_0), confidence(p_{n+1}))
            for e in eTrack do
                c_dist ← chebyshev_dist(p_0, eTrack_e)
                if c_dist < 5 then
                    conf ← ca − 0.05 ∗ c_dist
                    p_e ←(point(p_0), conf)
                else
                    conf ← ca − (0.25∗e)/n
                    p_e ← (point(eTrack_e), conf)
                end if
            end for
        else if dist < 10 then
            [p_1:p_n] ← (point(p_0), confidence(p_0))
        else
            [p_1:p_n] ← candidateTrack[p_1:p_n]
        end if
    end for
end for
```

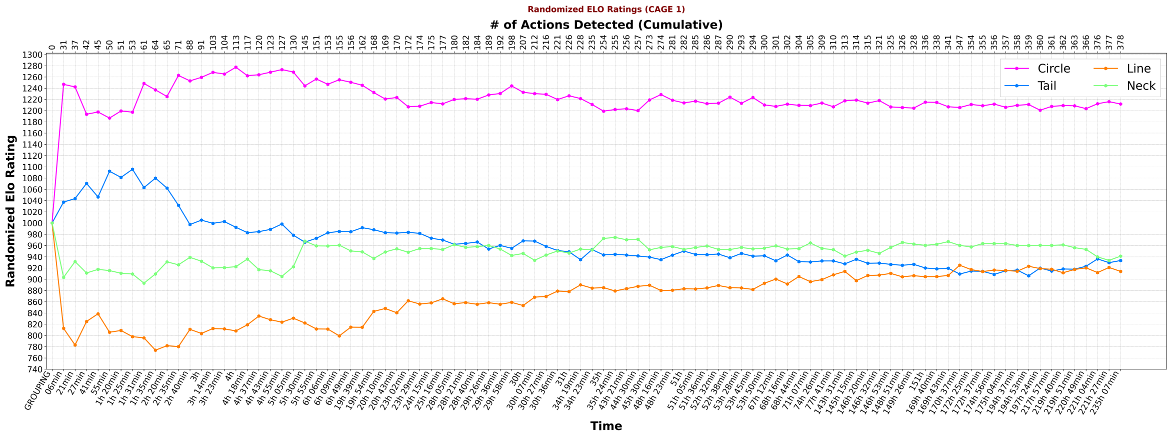

Supplementary Figure 2. Randomized Elo ratings of a group (#1) of 4 does (markings are indicated in the legend) over time representing the evolution of the hierarchy. The randomized Elo ratings were computed using Equation 3 with 1000 permutations.

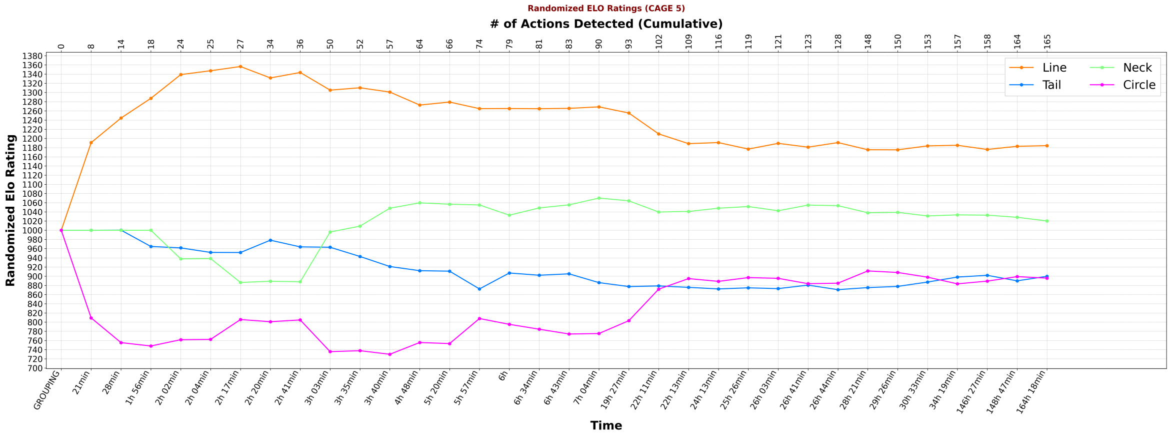

Supplementary Figure 3. Randomized Elo ratings of a group (#5) of 4 does (markings are indicated in the legend) over time representing the evolution of the hierarchy. The randomized Elo ratings were computed using Equation 3 with 1000 permutations.

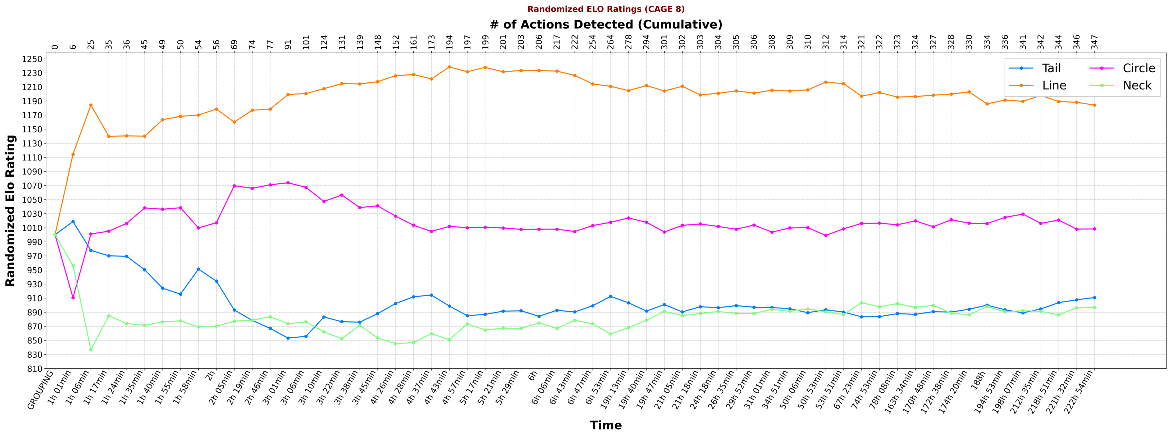

Supplementary Figure 4. Randomized Elo ratings of a group (#8) of 4 does (markings are indicated in the legend) over time representing the evolution of the hierarchy. The randomized Elo ratings were computed using Equation 3 with 1000 permutations.

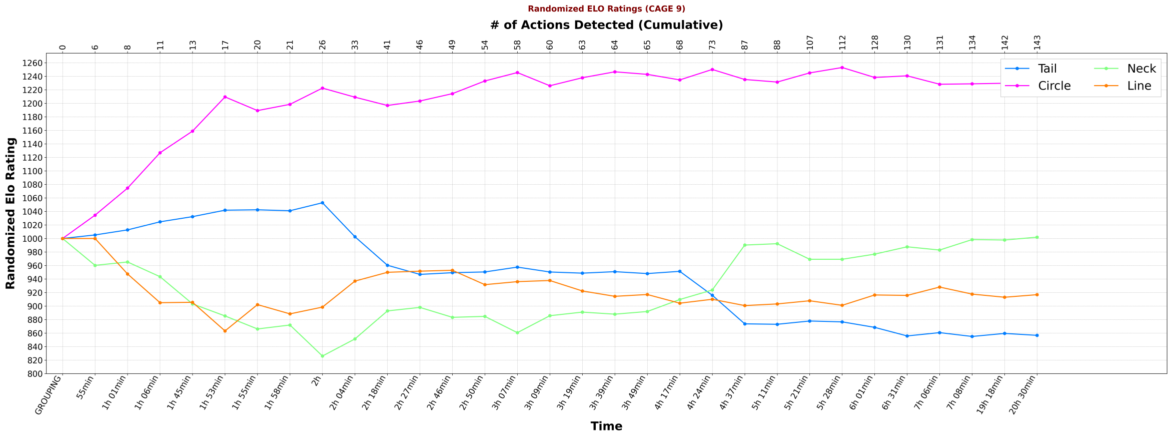

Supplementary Figure 5. Randomized Elo ratings of a group (#9) of 4 does (markings are indicated in the legend) over time representing the evolution of the hierarchy. The randomized Elo ratings were computed using Equation 3 with 1000 permutations.

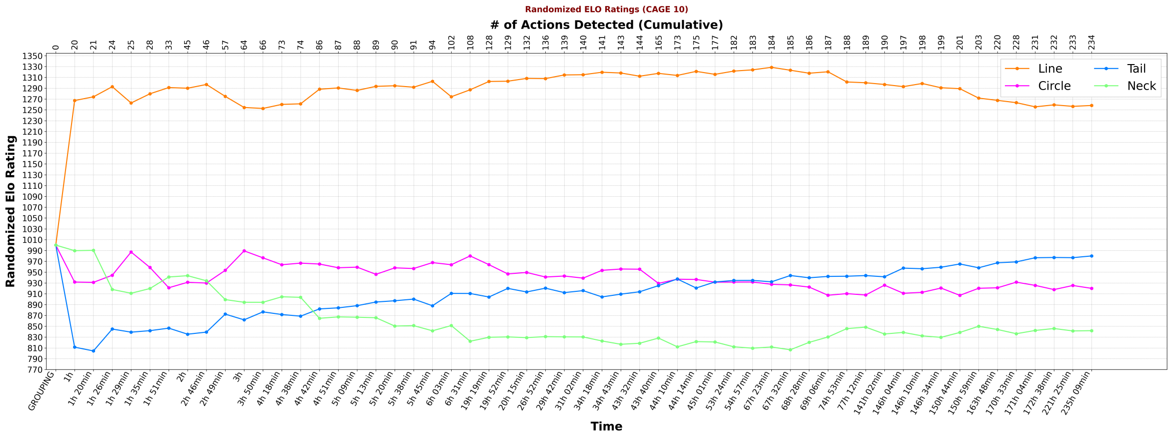

Supplementary Figure 6. Randomized Elo ratings of a group (#10) of 4 does (markings are indicated in the legend) over time representing the evolution of the hierarchy. The randomized Elo ratings were computed using Equation 3 with 1000 permutations.

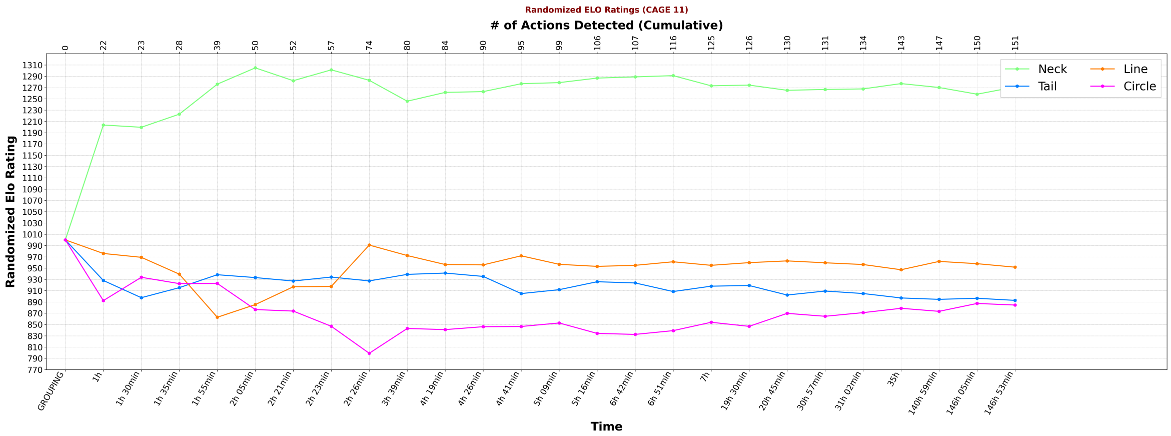

Supplementary Figure 7. Randomized Elo ratings of a group (#11) of 4 does (markings are indicated in the legend) over time representing the evolution of the hierarchy. The randomized Elo ratings were computed using Equation 3 with 1000 permutations.
